# Comparative Evaluation of GenoType MTBDR*plus* Line Probe Assay with Solid Culture Method in Early Diagnosis of Multidrug Resistant Tuberculosis (MDR-TB) at a Tertiary Care Centre in India

**DOI:** 10.1371/journal.pone.0072036

**Published:** 2013-09-05

**Authors:** Raj N. Yadav, Binit K. Singh, Surendra K. Sharma, Rohini Sharma, Manish Soneja, Vishnubhatla Sreenivas, Vithal P. Myneedu, Mahmud Hanif, Ashok Kumar, Kuldeep S. Sachdeva, Chinnambedu N. Paramasivan, Balasangameshwra Vollepore, Rahul Thakur, Neeraj Raizada, Suresh K. Arora, Sanjeev Sinha

**Affiliations:** 1 Department of Internal Medicine (Division of Research for Pulmonary, Critical Care & Sleep Medicine), All India Institute of Medical Sciences, New Delhi, India; 2 Department of Biostatistics, All India Institute of Medical Sciences, New Delhi, India; 3 Lala Ram Sarup Institute of Tuberculosis & Other Respiratory Diseases, New Delhi, India; 4 New Delhi TB Centre, State TB Training and Demonstration Centre, New Delhi, India; 5 Central TB Division, Ministry of Health & Family Welfare, Government of India, New Delhi, India; 6 Foundation for Innovative New Diagnostics (FIND), New Delhi, India; 7 State TB Office, New Delhi, India; Tulane University, United States of America

## Abstract

**Background:**

The objectives of the study were to compare the performance of line probe assay (GenoType MTBDR*plus*) with solid culture method for an early diagnosis of multidrug resistant tuberculosis (MDR-TB), and to study the mutation patterns associated with *rpoB, katG* and *inhA* genes at a tertiary care centre in north India.

**Methods:**

In this cross-sectional study, 269 previously treated sputum-smear acid-fast bacilli (AFB) positive MDR-TB suspects were enrolled from January to September 2012 at the All India Institute of Medical Sciences hospital, New Delhi. Line probe assay (LPA) was performed directly on the sputum specimens and the results were compared with that of conventional drug susceptibility testing (DST) on solid media [Lowenstein Jensen (LJ) method].

**Results:**

DST results by LPA and LJ methods were compared in 242 MDR-TB suspects. The LPA detected rifampicin (RIF) resistance in 70 of 71 cases, isoniazid (INH) resistance in 86 of 93 cases, and MDR-TB in 66 of 68 cases as compared to the conventional method. Overall (rifampicin, isoniazid and MDR-TB) concordance of the LPA with the conventional DST was 96%. Sensitivity and specificity were 98% and 99% respectively for detection of RIF resistance; 92% and 99% respectively for detection of INH resistance; 97% and 100% respectively for detection of MDR-TB. Frequencies of *katG* gene, *inhA* gene and combined *katG* and *inhA* gene mutations conferring all INH resistance were 72/87 (83%), 10/87 (11%) and 5/87 (6%) respectively. The turnaround time of the LPA test was 48 hours.

**Conclusion:**

The LPA test provides an early diagnosis of monoresistance to isoniazid and rifampicin and is highly sensitive and specific for an early diagnosis of MDR-TB. Based on these findings, it is concluded that the LPA test can be useful in early diagnosis of drug resistant TB in high TB burden countries.

## Introduction

MDR-TB poses a great threat to the TB control programmes worldwide [1]. India accounts for more than 25% of the world’s incident cases of tuberculosis [2]. There are an estimated 99,000 MDR-TB patients among incident total TB cases in India [3]. Early diagnosis of rifampicin (RIF) and isoniazid (INH) drug-resistant *Mycobacterium tuberculosis (Mtb)* is essential for efficient treatment and control of MDR-TB.

Solid and liquid culture methods for drug DST of *Mtb* are time consuming requiring weeks to months in providing the results. Further, contamination rates with conventional culture and DST are high. The World Health Organization (WHO), Geneva and Foundation for Innovative New Diagnostics (FIND), Geneva have endorsed the use of LPA test (GenoType MTBDR*plus,* Nehren, Germany) for rapid detection of MDR-TB directly from smear-positive sputum specimens and *Mycobacterium tuberculosis* cultures [4]. However, clinical utility of the test varies with the prevalence of particular mutations (incorporated in the test) in different geographical regions. There are limited data on the performance of this test from high TB burden countries including India. The LPA (MTBDR*plus*) detects mutations associated with the *rpoB* gene for RIF resistance, *katG* genes for high level INH resistance, and the *inhA* regulatory region gene for low-level INH resistance [5]. The aim of the study was to compare the performance of LPA test (GenoType MTBDR*plus* assay) with solid culture method among MDR-TB suspects for an early diagnosis of MDR-TB and monoresistance to isoniazid and rifampicin at a tertiary care centre in north India.

## Methods

### Study Subjects

The study was approved by the Institute Ethics Committee, All India Institute of Medical Sciences, New Delhi. Written informed consent was obtained from all patients. The patients were recruited from Chest Clinics of six districts in Delhi, medical outpatient department and DOT (Directly Observed Treatment) Centre of the All India Institute of Medical Sciences (AIIMS) hospital, New Delhi. All mycobacterial investigations were carried out at the Tuberculosis Laboratory of the Department of Internal Medicine. The laboratory is accredited for carrying out conventional DST and LPA tests by the National Mycobacteriology Accreditation System of Central TB Division, Ministry of Health and Family Welfare, Govt. of India. Sputum specimens from patients with a previous history of pulmonary TB treatment were subjected to microscopy. The smears were graded according to the number of bacilli seen on the slide, as per guidelines of the Revised National Tuberculosis Control Programme (RNTCP) of India [6]. All MDR-TB suspects from January to September 2012 with sputum bacillary load ≥1+ were enrolled in the study.

### Specimen Collection and Processing

Two sputum samples (spot and morning) were collected from each patient in 50 ml wide-mouthed sterile falcon tubes according to the revised National Guidelines [7]. Both sputum samples were subjected to smear examination and culture. The LPA test was done in one of the samples having a higher bacillary load. The sputum specimens were handled in class II biosafety cabinet in a bio-safety level (BSL)-3 laboratory and sputum specimens were decontaminated by N-acetyl-L-cysteine and sodium hydroxide (NALC-NaOH) method [8]. Subsequently, the sediments were suspended in 1–1.5 ml sterile phosphate buffer (pH 6.8) and two bottles of Lowenstein-Jensen medium were inoculated with each sample. 500 µl of the processed sample was used for DNA isolation in a screw capped tube.

### Conventional Drug Susceptibility Testing

The DST was carried out in Lowenstein-Jensen solid media by economic variant of 1% proportion method according to the standard operating procedure of RNTCP [9]. Rifampicin and isoniazid were tested with concentrations of 40 µg/ml and 0.2 µg/ml respectively. All isolates were identified as *Mycobacterium tuberculosis* by their slow growth rate, colony morphology, inability to grow on L-J media containing *p*-nitrobenzoic acid (500 µg/ml), and niacin positive and catalase negative tests. Any strain with 1% (the critical proportion) of bacilli resistant to any of the two drugs – rifampicin and isoniazid was classified as resistant to that drug.

### Line Probe Assay

**Figure 1:**
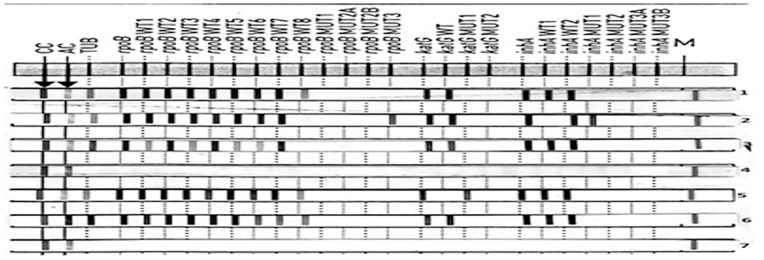
Representative patterns of line probe assay (GenoType MTBDR-plus) strip. Lane 1, susceptible to rifampicin (RIF) and isoniazid (INH); Lane 2, MDR- TB (*rpoB* S531L mutation and*inhA* C15T mutation); Lane 3, rifampicin monoresistant (mutation at rpoB530–533 gene region); Lane 4, absence of TUB band; Lan 5, isoniazid monoresistant (*katG* S315T1 mutation); Lane 6, DNA positive control (sensitive to rifampicin and isoniazid); Lane 7, DNA negative control.

The GenoType MTBDR*plus* line probe assay was performed according to the manufacturer’s (Hain Lifescience, Nehren, Germany) instructions. Three steps for LPA test included, DNA extraction, multiplex polymerase chain reaction (PCR) amplification and reverse hybridization. These steps were carried out in three separate rooms with restricted access and unidirectional workflow [10]. Mycobacterial DNA was extracted in BSL-3 laboratory according to manufacturer’s instructions. Briefly, 500 µl of decontaminated sputum sample was centrifuged at 10,000X*g* for 15 min, the supernatant was discarded and the pellet was resuspended in 100 µl sterile distilled water. The specimen was then heat killed at 95°C for 20 min in water bath. This was followed by sonication for 15 min and centrifugation at 13,000X*g* for 8 min. Five µl of the DNA supernatant was used for PCR while the remainder was stored at −20°C. Master mixture for amplification consisted of 35 µl primer nucleotide mixture (provided with kit), 5 µl of 10XPCR buffer with 15 mM MgCl_2_, 2 µl of 25 mM MgCl_2_, 0.2 µl (1 U) of HotStarTaq DNA polymerase (Hain Lifescience, Nehren, Germany), 3 µl nuclease free molecular grade water and 5 µl of DNA supernatant in a final volume of 50 µl. The amplification protocol consisted of 15 min of denaturation at 95°C, followed by 10 cycles comprising denaturation at 95°C for 30 sec and 58°C for 2 min. This was followed by 20 cycles comprising 95°C for 25 sec, 53°C for 40 sec and 70°C for 40 sec and a final extension at 70°C for 8 min. Hybridization was performed with the automatic machine (twincubator). After hybridization and washing, strips were removed, fixed on paper and results were interpreted [11]. Each strip of LPA had 27 reaction zones (bands), including six controls (conjugate, amplification, *M. tuberculosis* complex (TUB), *rpoB*, *katG* and *inhA* controls), eight *rpoB* wild-type (WT1–WT8) and four mutant probes (*rpoB* MUT D516V, *rpoB* MUT H526Y, rpo*B* MUT H526D, and *rpoB* MUT S531L), one *katG* wild-type and two mutant probes (*katG* MUT S315T1 and *katG* MUT S315T2), and two *inhA* wild type and four mutant probes (*inhA* MUT1 C15T, *inhA* MUT2 A16G, *inhA* MUT3A T8C, *inhA* MUT3B T8A) (Figure 1). Either missing of wild-type band or the presence of mutant band was taken as an indication of a resistant strain. Incomplete amplification of RIF and/or INH genes was considered as an invalid result. Laboratory staff performing conventional DST was unaware of LPA results, and vice versa. The turnaround time (hrs) was calculated from collection of samples to the availability of results.

### Statistical Analysis

Data were analysed by using Stata 11.2. Data were presented as frequency (percentage) and mean (SD). Sensitivity, specificity, negative predictive value, and positive predictive value with 95% confidence intervals were calculated. Categorical variables (like mutation group) were analysed by Chi square/Fisher’s exact test. P value less than 0.05 was taken as statistically significant.

## Results

A total of 269 previously treated sputum smear-positive patients (172 males and 97 females) were enrolled in the study. Their mean age was 34 (14, SD) yrs. All sputum samples were subjected to AFB microscopy, culture and DST by LJ and LPA methods. The smear examination yielded following bacillary load, 1+ AFB in 62 (23%), 2+ in 109 (41%) and 3+ in 98 (36%). Of 269 cultures, 251 (93%) were *Mycobacterium. tuberculosis*, 8 (3%) were culture negative, 3 (1%) demonstrated were non-tubercular mycobacteria (NTM), and the remaining 7 (3%) were contaminated (Table 1).

**Table 1:** *M.* *tuberculosis* detection by LPA vs. solid culture in 269 smear positive sputum samples.

	Solid culture method				
	Culture positive				
LPA Results	*Mtb*present	NTM	Culture negative	Contamination	Total
*M. tb* present	242	0	3	4	249
*M. tb* absent	2	3	1	1	7
Invalid	7	0	4	2	13
Total	251	3	8	7	269

LPA = Line probe assay. *M.tb* = *Mycobacterium tuberculosis*, NTM = Non-tubercular mycobacteria.

The LPA correctly identified *M. tuberculosis* in 242 of 251 *Mtb* culture positive samples (sensitivity 96%; 95% CIs, 93–98). The test demonstrated invalid results in 7 samples and two samples were *Mtb* negative (absence of TUB bands). All three sputum specimens having NTM also demonstrated absence of TUB bands on LPA tests (sensitivity, 100%; 95% CIs, 30–100).

For assessing the performance of LPA test, culture negative or contaminated samples, invalid LPA results or LPA test reports with absent TUB bands were excluded. Therefore, conventional phenotypic DST and valid genotypic LPA results were compared in 242 samples. Among these 242 *Mtb* culture positive samples, yield with phenotypic conventional DST was as follows; 68 (28%) were MDR-TB, 25 (10%) were INH monoresistant, another 3 (1%) were RIF monoresistant, while the remaining 146 (60%) were susceptible to both isoniazid and rifampicin.

Of the 68 MDR-TB results by conventional DST method, LPA detected 66 as MDR, one was RIF monoresistant and another one was INH monoresistant. Three samples with RIF monoresistance on conventional DST demonstrated similar results by LPA test. The LPA test demonstrated similar results in 19 of 25 samples with INH monoresistance and the remaining 6 samples were sensitive to both isoniazid and rifampicin. LPA yielded false genotypic resistance in two samples (1 RIF resistant and 1 INH resistant) which were phenotypically sensitive on LJ medium. Overall concordance between genotypic LPA test and phenotypic conventional DST was 96% (232/242).

Considering the phenotypic conventional Lowenstein-Jensen 1% proportion method as the gold standard, performance of GenoType MTBDR*plus* LPA in detecting resistance to rifampicin, isoniazid and MDR-TB is detailed in Table 2.

**Table 2:** Performance of LPA test as compared to LJ DST in detecting resistance to rifampicin, isoniazid, and MDR-PTB in 242 smear positive sputum samples.

	Rifampicin	Isoniazid	MDR-PTB
	Resistant N = 71,	Resistant N = 93,	Resistant N = 68,
	Sensitive N = 171	Sensitive N = 149	Sensitive N = 174
Sensitivity, %	98 (92–100)	92 (85–96)	97 (90–99)
Specificity, %	99 (97–100)	99 (96–100)	100 (98–100)
PPV, %	98 (92–100)	99 (94–100)	100 (94–100)
NPV, %	99 (97–100)	95 (91–99)	99 (96–100)

MDR-PTB = multi-drug resistant pulmonary tuberculosis; NPV = negative predictive value; PPV = positive predictive value,(values in parantheses are with 95% confidence intervals); 27/269 samples did not have both phenotypic and genotypic results and hence were excluded.

### Mutation Patterns in LPA

Among all 71 RIF-resistant strains, 51 (72%) [49/66 (74%) of MDR-TB strains and 2/5 (40%) of RIF- monoresistant strains] had a mutation in *rpoB* S531L (MUT3 band). This difference of *rpoB* S531L mutations in MDR-TB strains compared with RIF monoresistant strains was not statistically significant (p = 0.13). Other mutations associated with rifampicin resistance in MDR-TB strains included *rpoB* H526D (4/66), *rpoB* D516V (2/66) and *rpoB* H526Y (2/66), however, these three mutations were not seen in RIF monoresistant strains (Table 3).

**Table 3:** Band patterns of drug resistant *Mycobacterium tuberculosis* strains using line probe assay.

Gene	Band	Gene Region/Mutation	RIF Monoresistantstrains (n = 5)	INH Monoresistantstrains (n = 21)	MDR-PTB strains(n = 66)		P value
*rpoB*						RIF monoresistant Vs. MDR-TB	
	WT1	506–509	5(100)	21 (100)	66(100)	–	
	WT2	510–513	4(80)	21(100)	64(97)	0.199	
	WT3	513–517	5(100)	21(100)	62(94)	–	
	WT4	516–519	5(100)	21(100)	62(94)	–	
	WT5	518–522	5(100)	21(100)	66(100)	–	
	WT6	521–525	5(100)	21(100)	66 (100)	–	
	WT7	526–529	4(80)	21(100)	59(90)	0.4	
	WT8	53o- 533	2(40)	21(100)	10(15)	0.196	
	MUT1	D516V	0(0)	0(0)	2(3.0)	–	
	MUT2A	H526 Y	0(0)	0(0)	2(3.0)	–	
	MUT2B	H526 D	0 (0)	0(0)	4(6.0)	–	
	MUT3	S531 L	2 (40)	0(0)	49 (74)	0.13	
*katG*						RIF monoresistant Vs. MDR-TB	
	WT	315	5(100)	7(33.3)	9(14)	0.042	
	MUT1	S315 T1	0(0)	13(61.9)	60(90)	0.002	
	MUT2	S315T2	0(0)	0(0)	2(3.0)	–	
*inhA*							
	WT1	−15/−16	5(100)	14(67)	57(86)	0.042	
	WT2	−8	5(100)	21(100)	65(99)	–	
	MUT1	C15T	0(0)	6(28)	9(14)	0.1	
	MUT2	A16G	0(0)	0(0)	(0)	–	
	MUT3A	T8C	0(0)	0(0)	1(1)	–	
	MUT3B	T8A	0(0)	0(0)	0(0)	–	

RIF = rifampicin; INH = isoniazid; MDR-PTB = multidrug resistant-pulmonary tuberculosis. Percentage values are shown in parentheses.

The most frequent mutation found in INH resistant strains was *KatG* mutation [77/87 (88%)] which occurred more commonly in MDR-TB strains [62/66 (94%)] compared to INH monoresistant strains [15/21 (71%)]. Overall frequency of *inhA* mutation was 15/87 (17%) and was lower in MDR-TB strains [9/66 (14%)] as compared to INH monoresistant strains [6/21 (28%)]. This difference of mutations in MDR-TB strains compared with INH monoresistant was statistically significant for *KatG* gene (p = 0.01) but not for *inhA* gene (p = 0.12). Whereas, combined *KatG* and *inhA* mutation was found in 5/87 (6%) of MDR-TB strains, it was not seen in INH monoresistant strains. None of the single *inhA* MUT2 (A16G mutation), *inhA* MUT3A (T8C mutation) and *inhA* MUT3B (T8 Amutation) band was seen (Fig. 1).

Isolated *inhA* gene mutation was found in 4/66 (6%) of MDR-TB strains and 6/21 (28%) of INH monoresistant strains. The turnaround time of line probe assay was 48 hours; whereas, it was 70 days for phenotypic DST (28 days for conventional culture growth and another 42 days for DST).

## Discussion

In the recent years, a major emphasis has been given on rapid diagnosis and prompt initiation of accurate treatment of MDR-TB. Accurate and early diagnosis of MDR-TB is highly desirable as it interrupts further transmission of the disease and avoids empirical addition of life-saving drugs and thus amplification of drug resistance and creation of extensively drug resistant-tuberculosis (XDR-TB). It also avoids unnecessary cost of administration and occurrence of serious side - effects of second-line anti-tuberculosis drugs in case one is dealing with drug sensitive *M. tuberculosis* strains.

Present study was conducted in the mycobacteriology laboratory of a tertiary care centre in north India which is accredited for carrying out both DST on solid culture and LPA test. In this study, we evaluated performance of LPA test directly on sputum samples obtained from MDR-TB suspects. Patients were administered MDR-TB treatment based on LPA findings. Laboratory results were compiled later by a physician who was providing care to these patients. Subsequently, genotypic (LPA) and phenotypic (Lowenstein-Jensen proportion method) DST results were compared. We observed that the LPA test results had a good concordance with the conventional DST with a additional advantage of a shorter turnaround time.

In the present study, sensitivity (98%), specificity (99%) for detection of rifampicin resistance and specificity (99%) for detection of isoniazid resistance are in agreement with results of meta-analysis done by Ling et al [12]. However, a slightly higher sensitivity (92%) for detection of isoniazid resistance was observed in our study indicating that most of the mutations conferring INH resistance were found in the gene region that were incorporated in the present LPA strip [13]. Sensitivity (97%) and specificity (100%) for detection of MDR-TB in the present study corroborated with a previously reported study [14] and these findings suggest that performance of LPA is similar to conventional DST in a quality-assured TB laboratory. Sensitivity for detection of RIF resistance (inclusive of RIF monoresistance and RIF resistance in MDR-TB) with the LPA (MTBDR*plus*) test in the present study is comparable to that of GeneXpert MTB/RIF test, where detection of RIF-resistance in smear and culture positive sputum samples was 100% [15]. Currently, GeneXpert MTB/RIF test is the most rapid method for the diagnosis of MDR-TB with a turnaround time of approximately two hours. This test is based on real-time PCR method and the result of rifampicin resistance is used as a surrogate marker of MDR-TB; however, the test does not detect INH resistance and is likely to miss specimens with INH monoresistance. Hence, it can be inferred that among molecular tests LPA provides a better DST profile as compared to GeneXpert, and offers additional advantage of deciding the drug regimen in patients with INH monoresistance. WHO recommends addition of ethambutol as a third drug in the continuation phase in settings where the level of isoniazid resistance among new TB cases is high [16]. Additionally, this test can also be useful for systematic surveillance of INH monoresistance in countries with high isoniazid resistance.

In the present study, we encountered a major discrepancy in detecting INH resistance by genotypic method. The LPA test failed to detect INH resistant strains in 7 specimens, suggesting presence of some unidentified mutations in other genomic regions (like *ahpC, kasA, furA*) were not targeted by the assay used in the present study [17].

Rifampicin resistance is known to be associated with mutations in 81 base pair region (codon 527 to 533) of the *rpoB* gene [18], [19], [20]. The finding of dominant mutation for RIF resistance in *rpoB* S531L [51/71 (72%)], in the present study is similar to a previously published report [21]. One false RIF resistant strain with missing WT8 band was observed with the LPA test.

Distribution of mutations of *katG* and *inhA* genes is known to vary in different geographical regions. Frequencies of *katG* gene, *inhA* gene and combined *katG* and *inhA* gene mutations in the present study [72/87 (83%), 10/87 (11%) and 5/87 (6%) respectively] are within the range of previously reported studies [13], [22], [23], [24]. Finding of frequency of combined mutations of *KatG* and *InhA* in the present study is comparable to a recent study from Mumbai [25]. However, as compared to our study authors failed to find an association of *katG* and *inhA* genes mutations in INH monoresistant and MDR-TB strains.

Although there is no evidence of any relationship between specific mutation(s) conferring isoniazid resistance and treatment outcome; however, a recent study has reported better treatment outcomes in patients with *katG* mutations who received high-dose of isoniazid than those who received standard dose of the drug [26], suggesting clinical utility of detection of INH monoresistance pattern with the present LPA test.

The use of recent version of the assay in the present study provided an additional yield of INH resistance due to incorporation of *inhA* promoter region probes in the test [10/87 (11%); (4/66 (6%) in MDR-TB strains and 6/21 (28%) in INH monoresistant strains]. This could not have been detected by the previous version of line probe assay GenoType MTBDR as it lacked the *inhA* gene probe [27].

The present study highlights limitation of the conventional culture method because of valid LPA results in contamination [4/7; (57%)] or negative culture results [3/8; (38%)] and demonstrates superiority of LPA test. Majority of invalid LPA results in the present study were found in sputum specimens with lower bacillary load (1+), or culture negative samples, supporting the recommendation that the direct use of LPA test is not suitable for smear-negative clinical specimens [4], [28].

Limitations of Genotype MTBDR*plus* assay include need for an appropriate infrastructure, adequately trained and skilled laboratory personnel. The test is also not useful in sputum specimens with lower bacillary load and paucibacillary extrapulmonary TB specimens.

It is concluded from the present study that LPA test is highly sensitive and specific for rapid diagnosis of MDR-TB. The test detects resistance patterns with significantly lesser turnaround time as compared to conventional DST method. Additionally, the test also detects monoresistance to isoniazid and rifampicin. More studies with large number of samples are required to validate these preliminary findings, and define the exact place of this test in the diagnostic algorithm for MDR-TB under programmatic settings in high TB burden countries.

## References

[1] Sharma SK, Mohan A (2006) Multidrug-Resistant Tuberculosis: A menace that threatens to destabilize tuberculosis control. Chest 130: 261–272. 16840411 10.1378/chest.130.1.261

[2] World Health Organization (2012) Tuberculosis control in south east Asia region. The regional report 2012. SEA/TB/338. Geneva: WHO country office Thailand website. Available: http://whothailand.healthrepository.org/bitstream/123456789/1430/1/2012%20Report-Tuberculosis%20Control%20in%20SE%20Asia%20Region.pdf. Accessed 2013 July 15.

[3] World Health Organization (2010) Multidrug and extensively drug-resistant TB (M/XDR-TB): 2010 Global report on surveillance and response. WHO/HTM/TB/2010.3. Geneva (2010). WHO website. Available: http://apps.who.int/iris/bitstream/10665/44286/1/9789241599191_eng.pdf. Accessed 2013 July 15.

[4] World Health Organization (2008) Molecular line probe assays for rapid screening of patients at risk of multi-drug resistant tuberculosis (MDR-TB). Policy statement. Genewa (2008). TBC India website. Available: http://www.who.int/tb/features_archive/policy_statement.pdf. Accessed 2013 July 15.

[5] Hilleman D, Rusch-Gerdes S, Richter E (2007) Evaluation of the GenoType MTBDR*plus* assay for rifampin and isoniazid susceptibility testing of *Mycobacterium tuberculosis* strains and clinical specimens. J Clin Microbiol 45: 2635–2640. 17537937 10.1128/JCM.00521-07 PMC1951233

[6] Central TB Division, Directorate General of Health Services, Ministry of Health & Family Welfare, Government of India (2005) Revised National Tuberculosis Control Programme Laboratory Network (2005) Guidelines for quality assurance of smear microscopy for diagnosing tuberculosis. TBC India website.Available: http://www.tbcindia.nic.in/pdfs/RNTCP%20Lab%20Network%20Guidelines.pdf. Accessed 2013 July 15.

[7] Central TB Division, Directorate General of Health Services, Ministry of Health & Family Welfare, Government of India (2010) Revised National Tuberculosis Programme, DOTS-Plus Guidelines. TBC India website. Available: http://www.tbcindia.nic.in/pdfs/DOTS_Plus_Guidelines_Jan2010.pdf. Accessed 2013 July 15.

[8] Buyankhishig B, Oyuntuya T, Tserelmaa B, Sarantuya J, Lucero MG, et al. (2012) Rapid molecular testing for multi-resistant tuberculosis in mongoliya: A diagnostic accuracy study. Int J Mycobacteriol : 40–44. 10.1016/j.ijmyco.2012.01.007 26786948

[9] Central TB Division, Directorate General of Health Services, Ministry of Health & Family welfare, Government of India (2009) Revised National Tuberculosis Programme, Culture of *Mycobacterium tuberculosis* and drug susceptibility testing on solid medium. Manual of standard operating procedures. TBC India website. Available: http://www.tbcindia.nic.in/pdfs/standard%20operating%20procedures%20for%20C&DST%20labs.pdf. Accessed 2013 July 15.

[10] Albert H, Bwanga F, Mukkada S, Nyesiga B, Julius PA, et al (2010) Rapid screening of MDR-TB using molecular line probe assay is feasible in Uganda. BMC Infect Dis 10: 41. 20187922 10.1186/1471-2334-10-41 PMC2841659

[11] Anek-vorapong R, Sinthuwattanawibool C, Podewills LJ, McCarthy K, Ngamlert K, et al (2010) Validation of the GenoType® MTBDRplus assay for detection of MDR-TB in a public health laboratory in Thailand. BMC Infect Dis 10: 123. 20487550 10.1186/1471-2334-10-123 PMC2886057

[12] Ling DI, Zwerling AA, Pai M (2008) GenoType MTBDR assays for the diagnosis of multidrug-resistant tuberculosis: a meta-analysis. Eur Respir J 32: 1165–1174. 18614561 10.1183/09031936.00061808

[13] Raveendran R, Wattal C, Oberoi JK, Goel N, Datta S, et al (2012) Utility of GenoType MTBDR*plus* assay in rapid diagnosis of multidrug resistant tuberculosis at a tertiary care centre in India. Indian J Med Microbiol 30: 58–63. 22361762 10.4103/0255-0857.93034

[14] Huang WL, Chen HY, Kuo YM, Jou R (2009) Performance indicator assessment of the GenoType MTBDRplus test and DNA sequencing in detection of multidrug-resistant *Mycobacterium tuberculosis.* J Clin Microbiol. 47: 2520–2524. 10.1128/JCM.02499-08 PMC2725636 19494067

[15] Boehme CC, Naveta P, Hillemann D, Nicol MP, Shenai S, et al (2010) Rapid molecular detection of tuberculosis and rifampin resistance. N Eng J Med 363: 1005–1015. 10.1056/NEJMoa0907847 PMC2947799 20825313

[16] World Health Organization (2010) Treatment of tuberculosis. Guidelines. Geneva (2010). WHO/HTM/TB/2009.420. WHO website. Available: http://whqlibdoc.who.int/publications/2010/9789241547833_eng.pdf. Accessed 2013 July 15.

[17] Silva PE, Palomino JC (2011) Molecular basis and mechanisms of drug resistance in *Mycobacterium tuberculosis*. Classical and new drugs. J Antimicrob Chemother 66: 1417–1430. 21558086 10.1093/jac/dkr173

[18] Mani C, Selvakumar N, Narayanan S, Narayanan PR (2001) Mutations in *rpoB* gene of multidrug-resistant *Mycobacterium tuberculosis* clinical isolates from India. J Clin Microbiol 39: 2987–2990. 11474030 10.1128/JCM.39.8.2987-2990.2001 PMC88277

[19] Cavusoglu C, Turhan A, Akinci P, Soyler I (2006) Evaluation of the GenoType MTBDR Assay for rapid detection of rifampin and isoniazid resistance in *Mycobacterium tuberculosis* isolates. J Clin Microbiol 44: 2238–2242. 10.1128/JCM.00425-06 PMC1489470 16825346

[20] Yue J, Shi W, Xie J, Li Y, Zeng E, et al (2003) Mutations in the *rpoB* gene of multidrug-resistant *Mycobacterium tuberculosis* isolates from China. J Clin Microbiol 41: 2209–2212. 12734282 10.1128/JCM.41.5.2209-2212.2003 PMC154692

[21] Miotto P, Piana F, Penati V, Canducci F, Migliori GB, et al (2006) Use of GenotypeMTBDR assay for molecular detection of rifampicin resistance in *Mycobacterium tuberculosis* clinical strains isolated in Italy. J Clin Microbiol 44: 2485–2491. 16825369 10.1128/JCM.00083-06 PMC1489497

[22] Tukvadze N, Kempker RR, Kalandadze L, Kurbatova E, Leonard MK, et al (2012) Use of molecular diagnostic test in AFB smear positive tuberculosis suspects greatly reduces time to detection of multidrug-resistant tuberculosis. PLoS One 7(2): e31563. 22347495 10.1371/journal.pone.0031563 PMC3276512

[23] Barnard M, Albert H, Coetzee G, O’Brien R, Bosman ME (2008) Rapid molecular screening for multidrug-resistant tuberculosis in high-volume public health laboratory in South Africa. Am J Respir Crit Care Med 177: 787–792. 18202343 10.1164/rccm.200709-1436OC

[24] Kiepiela P, Bishop KS, Smith AN, Roux L, York DF (2000) Genomic mutations in the katG, inhA and aphC genes are useful for the prediction of isoniazid resistance in *Mycobacterium tuberculosis* isolates from Kwazulu Natal, South Africa. Tuber Lung Dis 80: 47–56. 10897383 10.1054/tuld.1999.0231

[25] Tolani MP, Dsouza DT, Mistry NF (2012) Drug resistance mutations and heteroresistance detected using the GenoTypeMTBDR*plus* assay and their implication for treatment outcomes in patients from Mumbai, India. BMC Infect Dis 12: 9. 22260344 10.1186/1471-2334-12-9 PMC3283446

[26] Jacobson KR, Theron D, Victor TC, Streicher EM, Warren RM, et al (2011) Treatment outcomes of Isoniazid resistant tuberculosis patients, western cap province, South Africa. Clin Infect Dis 53 (4): 369–372. 10.1093/cid/cir406 PMC3202325 21810750

[27] Causse M, Ruiz P, Zerolo J, Casal M (2008) Evaluation of new GenoTypeMTBDR*plus* for detection of resistance in cultures and direct specimens of *Mycobacterium tuberculosis.*Int J Tuberc Lung Dis. 12: 1456–1460. 19017457

[28] Dorman SE, Chihota VN, Lewis JJ, Meulen MVD, Mathema B, et al (2009) GenoType MTBDRplus for direct detection of *Mycobacterium tuberculosis* and drug resistance in strains from gold miners in south Africa. J Clin Microbiol 50: 1189–1194. 10.1128/JCM.05723-11 PMC3318561 22238443

