# Correction: Comparative Evaluation of GenoType MTBDR*plus* Line Probe Assay with Solid Culture Method in Early Diagnosis of Multidrug Resistant Tuberculosis (MDR-TB) at a Tertiary Care Centre in India

**DOI:** 10.1371/annotation/e90efdb7-91c1-45f0-ae98-a88fcb407acc

**Published:** 2013-10-10

**Authors:** Raj N. Yadav, Binit K. Singh, Surendra K. Sharma, Rohini Sharma, Manish Soneja, Vishnubhatla Sreenivas, Vithal P. Myneedu, Mahmud Hanif, Ashok Kumar, Kuldeep S. Sachdeva, Chinnambedu N. Paramasivan, Balasangameshwra Vollepore, Rahul Thakur, Neeraj Raizada, Suresh K. Arora, Sanjeev Sinha

The affiliation given for the third author, Surendra K. Sharma, was incorrect. The correct affiliation for this author is available below.

Department of Medicine, All India Institute of Medical Sciences, New Delhi, India 

